# The type of ploidy of chrysanthemum is not black or white: a comparison of a molecular approach to published cytological methods

**DOI:** 10.3389/fpls.2014.00479

**Published:** 2014-09-23

**Authors:** Maik Klie, Stephan Schie, Marcus Linde, Thomas Debener

**Affiliations:** ^1^Department of Molecular Plant Breeding, Institute for Plant Genetics, Leibniz Universität HannoverHannover, Germany; ^2^Kleinwanzlebener Saatzucht AG formerly Rabbethge and GieseckeEinbeck, Germany; ^3^Saaten-Union Biotec GmbHLeopoldshöhe, Germany

**Keywords:** allopolyploidy, autopolyploidy, molecular marker, polysomic inheritance, single dose markers, linkage in repulsion

## Abstract

Polyploidy is a widespread phenomenon among higher plants and a major factor shaping the structure and evolution of plant genomes. The important ornamental chrysanthemum (*Chrysanthemum indicum* hybrid) possesses a hexaploid genome with 54 chromosomes and was classified based on its evolutionary origin and cytological methods as an allopolyploid. However, it is questionable whether cytological methods are sufficient to determine the type of ploidy, and there are more informative methods available based on molecular marker analyses. Therefore, we collected segregation data for 406 dominant molecular marker alleles [327 amplified fragment length polymorphism (AFLPs), 65 single-strand conformation polymorphism (SSCPs) and 14 microsatellites (EST-SSRs)] in a biparental F1 population of 160 individuals. We analyzed these data for the characteristics that differ between allopolyploids and autopolyploids, including the segregation ratio of each marker, the ratio of single-dose (SD) to multi-dose (MD) markers, the ratio of SD markers in coupling to those in repulsion and the banding patterns of the SSRs. Whereas the analysis of the segregation ratio of each polymorphic marker indicated disomic (13 markers) as well as hexasomic (eight markers) inheritance, the ratio of SD markers in coupling to those in repulsion was 1:0, which is characteristic of autopolyploids. The observed ratio of SD to MD markers was 0.67:0.33 which is significantly different to the expected segregation for auto- and allohexaploids. Furthermore, the three EST-SSR alleles were inherited in all possible combinations and were not independent of each other, as expected for fixed heterozygosity in allopolyploids. Combining our results with published cytological data indicates that cultivated chrysanthemums should be classified as segmental allohexaploids.

## INTRODUCTION

Chrysanthemums (*Chrysanthemum indicum* hybrid, *C.* x *grandiflorum* or *C. morifolium*) are among the most economically important ornamental plants worldwide and are produced as cut flowers and as potted or garden plants. Chrysanthemums belong to the large plant family *Asteraceae* and are native to the Northern Hemisphere, primarily Europe and Asia (Dowrick, 1952). Cultivated chrysanthemums are generally believed to be the result of natural hybridization involving several different species, such as *C. indicum* L., *C. morifolium*, *C. vestitum,* and *C. lavandulifolium* (Vogelmann, 1969; Dai et al., 1998; Yang et al., 2006). These crosses led to the formation of a hexaploid hybrid complex with 54 chromosomes (Dowrick, 1953).

Because cultivated chrysanthemums resulted from hybridization events between different species, and because the occurrence of bivalent chromosomes is detected in meiosis in all four investigated polyploid *Chrysanthemum* accessions (Watanabe, 1977; Li et al., 2011), the cultivated forms are currently classified as allohexaploids. However, polyploid genomes can be highly dynamic, and Stebbins (1947) proposed that it might be difficult to unambiguously classify the type of ploidy of an organism. This was also indicated by Watanabe (1983) for the hexaploid *C. japonense*, which is not believed to be a progenitor of the *C. indicum* hybrid, reporting a very limited formation of multivalents (3.8%) using microscopic methods. In contrast, Watanabe (1977) and Li et al. (2011) state a clear autopolyploid behavior in cytological studies of *Chrysanthemum* species closely related to the ornamental types. Therefore, it is necessary to combine cytological and molecular methods to clarify the type of ploidy.

Polyploids are classified into the two major categories of auto- and allopolyploids. Allopolyploids are characterized by preferential pairing of chromosomes or fixed heterozygosity, which results from the combination of divergent parental genomes, bivalent chromosome formation in meiosis and disomic inheritance at each locus. In contrast, for autopolyploids the formation of multivalent chromosomes and polysomic inheritance is generally assumed (Stebbins, 1947; Soltis and Soltis, 2000). However, in addition to these extremes, intermediary forms have also been described (Stebbins, 1947; Sybenga, 1969).

In addition to cytological methods, Wu et al. (1992) described the usefulness of single-dose (SD) molecular markers to distinguish allopolyploidy from autopolyploidy. SD markers are characterized by only one dominant marker allele at a single locus and can be distinguished from MD markers by determining the means of the corresponding recombination frequencies (Mather, 1957). Silva et al. (1993) determined the theoretical ratios of SD to MD markers for allo- and autopolyploids, which might indicate the ploidy type of an organism. A SD marker present in only one parent (uniparental marker) has a theoretical segregation ratio of 1:1 (presence: absence) in an F1 progeny of both autopolyploids and allopolyploids. Likewise, biparental markers will segregate in a 3:1 (presence:absence) ratio in both auto- and allopolyploids. In contrast, MD markers have more complex segregation ratios that differ between autopolyploids and allopolyploids. The expected ratios for SD to MD markers is 0.625:0.375 in allopolyploids and 0.75:0.25 in autopolyploids (Silva and Sorrells, 1996) so that the type of ploidy can be inferred if a larger number of markers is tested for SD versus MD segregation.

Furthermore, Wu et al. (1992) used SD markers for 75 individuals and showed a linkage in the coupling phase for allo- and autopolyploids, whereas a linkage in the repulsion phase can be detected only in allopolyploids. By calculating the ratio of markers in coupling to those in repulsion, it is possible to distinguish allopolyploidy (ratio of 1:1) from autopolyploidy (ratio of 1:0 for polyploids above tetraploidy). Additionally, the banding patterns of sequence specific markers, such as SSRs, reflect the distributions of the homologous and homeologous chromosomes within the progeny. Thus, this type of marker is informative in determining the pairing of the chromosomes, as it indicates the occurrence of fixed heterozygosity and therefore also the type of ploidy.

By using molecular markers, we sought to determine whether the classification of chrysanthemum as allo-hexaploid, based on cytological methods, is conclusive. Knowledge about the type of ploidy is of interest from an exploratory and a breeder’s point of view because desirable alleles cannot be freely combined in allo-hexaploid genotypes. Therefore, we describe the use of amplified fragment length (AFLP), single-strand conformation polymorphism (SSCP) and microsatellite (SSR) markers in a segregating biparental F1 population to investigate the type of ploidy of cultivated chrysanthemums. Additionally, we compare our results with previously published cytological data.

## MATERIALS AND METHODS

### PLANT MATERIAL

We established a segregating biparental F1 population (MK11/3) of 160 individuals by crossing the female parent *C. indicum* hybrid “Kitam” (541) with the paternal parent “Relinda” (VZR), which is a registered *C. indicum* hybrid variety. Three cuttings of each genotype were cultivated with 48 plants per m^2^ in plots of 12.5 cm × 12.5 cm. The plants were grown in a fertilized substrate (a mixture of peat moss and chalked compost soil) in a greenhouse under a 16 h light/8 h dark cycle at a constant temperature of 22°C.

### DNA EXTRACTION

For the DNA extraction, 70 mg of unfolded, young leaves was used. The plant material was dried overnight at 37°C, frozen in liquid nitrogen and ground using a bead mill. The extraction was performed using the NucleoSpin Plant II Kit from Macherey and Nagel (Düren, D) following the manufacturer’s instructions, with minor modifications. The concentration of genomic DNA was assessed spectrophotometrically at 260 nm and was evaluated for purity by determining the OD 260 nm/280 nm and the OD 260 nm/230 nm ratios. The DNA quality was assessed by agarose gel electrophoresis.

### MARKER ANALYSIS

#### AFLP analysis

The AFLP analysis was performed as described previously (Vos et al., 1995), with minor modifications according to Klie et al. (2013). For each sample, 100 ng of DNA was digested with 9 U *Hind*III (Fisher Scientific – Germany GmbH, Schwerte, D) and 3.5 U *Mse*I (Fisher Scientific - Germany GmbH, Schwerte, D). The preamplification reactions were performed with specific primers that had an A as a selective base at the 3′ end [*Hind*III (5′-AGACTGCGTACCAGCTT-A-3′) and *Mse*I (5′-GACGATGAGTCCTGAGTAA-A-3′)]. *Hind*III (5′-AGACTGCGTACCAGCTT-ANN-3′) primers with two extra selective bases and *Mse*I (5′-GACGATGAGTCCTGAGTAA-ANNN-3′) primers with three extra selective bases were used for the final amplification. The *Hind*III primers were end-labeled with an infrared dye (either IRD 700 or IRD 800; Eurofins MWG, Ebersberg, D). In a single PCR reaction, labeled primers were used either as single primers or in combinations of two differently labeled primers (IRD 700 and IRD 800). In total, 21 selective primer combinations were analyzed (**Table 1**). The fragments were separated on 6 % polyacrylamide gels (Sequagel XR, Hessle, UK) using a DNA analyzer (LI-COR, Lincoln, Nebraska, USA) and automatically processed using the e-Seq-Software (V3.0, LI-COR, Lincoln, Nebraska, USA).

**Table 1:** The primer combinations used for the amplified fragment length polymorphism (AFLP) analysis.

*Hind*III- IRD 700	*Hind*III- IRD 800	*Mse*I
AGC	AGT	ACCG
AAT	AGT	ACAG
AAT	ACG	ATGG
AGC	ACA	ACAT
AAT	–	ACGA
AGA	–	ACGG
AGT	–	ATAG
AAC	–	ACCT
AAT	–	ATGA
–	ACA	AAGC
–	ACG	AGCA
–	ACG	AAGC
–	ACG	ACGA
–	ACG	ACAC
–	ACG	ATCA
–	ACA	ACCA
–	ACA	ACAG

*Only the selective bases are listed in the table below. The framework of the selective HindIII primers was 5′-AGACTGCGTACCAGCTT-NNN-3′, and that of the selective MseI primers was 5′-GACGATGAGTCCTGAGTAA-NNNN-3′.*

#### SSCP markers for candidate genes

Mutant screens in *Arabidopsis* and other plants identified several genes that control shoot branching and are involved in strigolactone biosynthesis and perception. Some of these genes, such as *CCD*8 (Liang et al., 2010), *MAX*2 (Dong et al., 2013) and *BRC*1 (Chen et al., 2013), have also been characterized in chrysanthemum. In addition, we isolated a *CCD*7 homolog from chrysanthemum (unpublished) and screened this sequence and those of the other genes containing polymorphisms using SSCP analysis. Several primer pairs were used that covered various fragments of each candidate gene (**Table 2**). Most of the PCR products were IRD-labeled using the universal M13 sequences (5′-GTAAAACGACGGCCAGT-3′ for the forward primer and 5′-CAGGAAACAGCTATGAC-3′ for the reverse primer) at the 5′ end (Schuelke, 2000). The PCR conditions were as follows: 0.2 μM of each unlabeled primer, 0.07 μM of each labeled primer and 0.07 μM of a M13 primer end-labeled with either the IRD 700 dye or the IRD 800 dye (Eurofins MWG, Ebersberg, D) in a final 25 μL reaction volume [2x Williams Buffer, 0.16 mM dNTPs, 0.7 U DCS-Taq polymerase (Enzymatics, Beverly, MA, USA) and 30 ng template DNA]. The conditions of the PCR amplification were as follows: 95°C (3 min), then 25 cycles at 94°C (30 s)/58°C (30 s)/72°C (45 s), followed by eight cycles at 94°C (30 s)/52°C (45 s)/72°C (60 s), and a final extension at 72°C for 10 min. All other PCR products, which were visualized by silver staining according to the protocol of Sanguinetti et al. (1994), were amplified by a standard PCR reaction in a final reaction volume of 20 μl containing 1x Williams Buffer, 0.2 mM dNTPs, 0.5 μM primers, 0.5 U DCS Taq polymerase and 30 ng template DNA. The conditions of the PCR amplification were as follows: 95°C (3 min), then 30 cycles at 94°C (30 s)/60°C (60 s)/72°C (60 s), followed by a final extension at 72°C for 10 min. An equal volume of SSCP dye (95 % formamide, 0.01 M NaOH, 0.05% xylene cyanol, and 0.05% bromophenol blue) was added to each PCR reaction, and this step was followed by denaturing the samples for 3 min at 95°C. The denatured samples were immediately placed on ice prior to loading onto cooled (10°C) 0.5x MDE gels [0.5x MDE^®^ gel solution (Lonza Group Ltd., Basel, SUI), 0.6x long run TBE (80.4 mM Tris, 7.5 mM boric acid, and 1.5 mM EDTA), 8.3% glycerine, 0.05% APS, 10 μl TEMED and *ad* 15 ml water]. IRD-labeled single strands were detected with the Odyssey^®^ Infrared Imaging System (LI-COR, Lincoln, Nebraska, USA) and automatically documented using Odyssey Software (V3.0, LI-COR, Lincoln, Nebraska, USA). The non-IRD-labeled single strands were visualized by silver staining according to the protocol of Sanguinetti et al. (1994).

**Table 2:** A list of the primer pairs for the candidate genes *CCD*7, *CCD*8, *MAX*2, and *BRC*1 used in the single-strand conformation polymorphism (SSCP) analysis.

Gene	Accession	Primer pairs		Product size	Detection method
CCD7	unpublished	F	CCCTCTAGATGGTCATGG	550 bp	Silver staining
		R	AGCAAGATCTAACAAGTCCACACCAC		
		F*	TGTCATGCAACGCAGAGGAT	1750 bp	M13-IRD700
		R	CCCACATTTGAGAAGGAGCTT		
		F	GGTGGGGCCCCTTACGAGAT	600 bp	Silver staining
		R	GCATTGCATGACATCATAAG		
		F*	TCCATGACTGGGCTTTCACA	380 bp	M13-IRD700
		R	CCCACATTTGAGAAGGAGCTT		
CCD8	Liang et al. (2010)	F*	ATGGCATCCTGAGTCGAAAG	550 bp	M13-IRD700
		R	GCGTCTACTAGTTCTCCCTTTGG		
		F*	ACAAGCTGCGGCTTCAAA	260 bp	M13-IRD700
		R	GCGTCTACTAGTTCTCCCTTTGG		
		F*	GGTGCGTCCCTAACTGACAA	480 bp	M13-IRD700
		R	GACTCAGGATGCCATTCAAAC		
MAX2	JX556222	F*	GCCAATCCAGGGTCGGATAC	550 bp	M13-IRD700
		R	GTAACGACAAACTCCTCTGG		
		F*	ATGTCTTTCTCCACCACAACAAT	1400 bp	M13-IRD700
		R	AAGCCTACTCGCACTCAACG		
BRC1	JX870411	F	TGCAGCATCAGTTCAGTGACT	380	M13-IRD700
		R*	AGCAGTAGCATACAATTGACATAGT		

*The gene, gene bank accession (if available), primer sequence (5′–3′), size of expected PCR product and detection method are given. Primers marked by an asterisk contained a universal M13 sequence (5′-GTAAAACGACGGCCAGT-3′ for forward primers or 5′-CAGGAAACAGCTATGAC-3′ for reverse primers) at the 5′ end for infrared (IRD) labeling of the PCR fragments. Those fragments were detected via infrared imaging, whereas non-labeled fragments were detected via silver staining.*

#### EST-SSR markers

Sequence information for 7009 ESTs from *Chrysanthemum morifolium* was downloaded from NCBI (November 2010). These ESTs were screened for mono-, di-, tri-, tetra-, penta-, hexa-, and hepta-nucleotide motifs of microsatellites with a copy number of at least four repeats using the tandem repeat finder (Benson, 1999). For the 21 SSR-containing ESTs, primer pairs were designed using the Primer3Plus software (Untergasser et al., 2007) with the default settings. Each forward primer was extended by a universal M13 sequence tag (5′-GTAAAACGACGGCCAGT-3′) at the 5′ end for IRD-labeling of the PCR fragments (Schuelke, 2000). The three EST-SSR markers (**Table 3**) were used on the entire population using the PCR conditions as described previously. The PCR products were separated on 6% polyacrylamide gels (Sequagel XR, Hessle, UK) using a DNA Analyzer (LI-COR, Lincoln, NE, USA) and automatically documented using e-Seq-Software (V3.0, LI-COR, Lincoln, Nebraska, USA).

**Table 3:** List of the three polymorphic EST-SSR markers used on the chrysanthemum MK11/3 population.

Accession	Forward primer	Reverse primer	Product size	Motif	Copy number
69838459	CCTCTCCTCCCAACAAACAA	CCGTAAGTGCCTTCACCAAT	209 bp	AAG	8
69834897	CCGCTACAATTCAAACAAACAA	GTGGTGGTGGTTGAGAACCT	207 bp	AATCCA	5
69837400	CCAATTGAGGCGTTTTGTTT	CATTTTCCACGTAAGCACCA	239 bp	GGT	10

*The GB accession of the chrysanthemum EST, primer sequence (5′–3′), size of the expected PCR product, motif and number of repeats are given.*

### DATA ANALYSIS

The marker banding patterns for each genotype were visually scored as present (1), absent (0), or ambiguous (?).

According to Mather (1957), the uniparental and biparental markers were classified as SD or MD markers using the geometric means between the two segregation distributions. For the uniparental markers, the geometric mean was calculated between the 1:1 and the 3:1 distribution by the formula 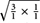 = 1.73 as the point for selection, whereas for the biparental markers, the mean between the 3:1 and 15:1 distribution was determined by the equation 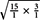 = 6.71 for selection (Grivet et al., 1996). For each marker, the segregation ratio was estimated and compared to the corresponding selection point. Markers with ratios below this point were classified as SD markers, and those with ratios above the threshold were classified as MD markers. Silva et al. (1993) estimated the theoretical proportion of SD to MD markers as 0.625–0.375 for allopolyploidy and and 0.75 to 0.25 for autopolyploidy in hexaploids. We compared our calculated ratios to these ratios using the chi-square test in the R software (version 2.15.2, R Core Team, 2012).

By determining the ratio of SD markers in coupling to those in repulsion in a population of 75 individuals, Wu et al. (1992) distinguished allopolyploidy (ratio of 1:1) from autopolyploidy (ratio of 1:0). We estimated this ratio using the previously selected uniparental SD markers of the MK11/3 population for each parent. We generated linkage maps with a maximal recombination frequency of 0.35 for 75 randomly selected offspring in the backcross-1 (BC1) mode of JoinMap version 4 (Van Ooijen, 2006). The markers were placed into linkage groups based on their independent LOD values, which ranged from 4 to 10. The marker distances in centimorgans were calculated using Kosambi’s mapping function. Subsequently, the values of the marker data matrix were inverted so that the present bands were coded as absent and the absent bands were coded as present. These inverted markers were integrated into the previously calculated maps. The markers that were linked in the original dataset were designated to be in coupling, and the markers that showed linkage between the original and the inverted datasets were designated to be linked in repulsion (Ukoskit and Thompson, 1997; Kriegner et al., 2003). The resulting ratio of markers in the coupling to the repulsion phase was compared to the assumed ratios (Wu et al., 1992) for allopolyploidy (1:1) and autopolyploidy (1:0 for polyploids above tetraploidy) using the chi-square test in the R software (version 2.15.2, R Core Team, 2012).

## RESULTS

### MOLECULAR MARKER DATA FOR THE MK11/3 POPULATION

Allo- and autohexaploids differ in their segregation ratios, their ratios of marker dosage and their ratios for markers in coupling to those in repulsion. Therefore, we used various molecular markers, such as AFLP, SSCP, and SSR markers, to investigate the inheritance patterns in chrysanthemum.

All of the segregating marker fragments were analyzed dominantly because of the complex banding patterns for even single-locus markers, such as SSR or SSCP markers, in a hexaploid genome. In total, 406 polymorphic markers were scored in the MK11/3 population. The vast majority were AFLP markers with 327 fragments derived from 21 primer combinations, followed by 65 SSCP marker fragments for the candidate genes *CCD*7 (29 fragments), *CCD*8 (16 fragments), *MAX*2 (8 fragments), and *BRC*1 (12 fragments) and 14 DNA fragments derived from the three EST-SSRs.

Marker segregation types of 1:0 or 7:1 are expected for an allopolyploid organism, and types of 4:1 or 9:1 are expected for an autopolyploid organism. Accordingly, all of the polymorphic markers were tested to determine whether their segregation ratios were consistent with autopolyploidy or allopolyploidy by the chi-square test (**Table 4**). For 204 of the total 406 markers significant possible segregation types were assigned by the statistical test. Not all markers could be assigned because a large number of individuals is needed to clearly distinguish between different segregation types (e.g., a hexasomic 15:1 or a disomic 24:1 segregation). The 1:1 segregation pattern does not distinguish between the types of ploidy and is therefore not informative. A large proportion of the markers (34) displayed a skewed segregation and did not fit to any of the ratios diagnostic for allo- or autopolyploidy. In total, 13 markers segregated in a disomic manner, with four, four and two of them linked to each other, whereas eight markers showed a 4:1 ratio that is characteristic of hexasomic inheritance. Therefore, there are more markers indicating a disomic inheritance, as expected for an allo-hexapolyploid genome.

**Table 4:** The marker segregation types for the MK11/3 population.

Parental composition	Segregation ratio	Number of markers	Type of segregation
Maternal	1:1	63	Non-informative
Paternal	1:1	85	Non-informative
Maternal	1:2	17	Skewed
Paternal	1:2	18	Skewed
Maternal	3:1	7	Disomic, duplex × nulliplex
Paternal	3:1	5	Disomic, duplex × nulliplex
Maternal	4:1	3	Hexasomic, duplex × nulliplex
Paternal	4:1	5	Hexasomic, duplex × nulliplex
Biparental	7:1	1	Disomic, duplex × simplex

*Only the 204 markers that were assigned a segregation ratio expected for uniparental and biparental markers by the chi-square test (1-α = 0.95; *df* = 1) are shown.*

### THE SEGREGATION PATTERNS OF SSR MARKER FRAGMENTS

**FIGURE 1:**
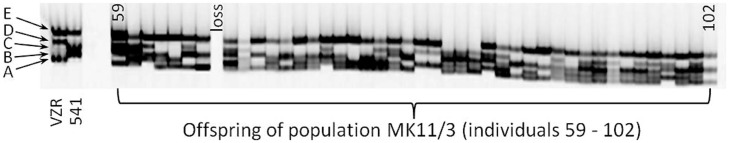
**The segregation pattern of EST-SSR 69834897 for selected individuals from the MK11/3 population.** Different genotypes are represented by individual lanes. The maternal parent 541 and the paternal parent VZR are shown on the left side of the gel. The three alleles of each parent are indicated as A to E, with E being present in both genotypes.

The three SSR markers amplified four (marker 69838459) or five (markers 69834897 and 69837400) fragments. An example of the segregation pattern of the EST-SSR marker 69834897 is given in **Figure 1**. This marker amplified five alleles in the progeny MK11/3, of which four (A–D) were polymorphic between the maternal (541) and paternal parents (VZR). For all three EST-SSRs, an independent assortment of the amplified alleles was observed, as expected for polysomic inheritance. No cosegregation of specific alleles was observed, nor was any allele combination found to exclude another, as would be expected in the case of disomic inheritance with fixed heterozygosity in allopolyploids.

### MARKER DOSAGE RATIOS

Of the 406 segregating uni- and biparental markers, 273 were classified as SD markers and 133 were classified as MD markers according to their segregation ratios (Mather, 1957). The ratio of SD to MD markers was estimated to be 0.67–0.33 and was compared to the theoretical proportion of SD to MD markers indicative of auto- and allopolyploidy (Silva et al., 1993; Silva and Sorrells, 1996; Ukoskit and Thompson, 1997) using the chi-square test (**Table 5**). The ratio was significantly different from the expected ratio for allopolyploidy and for autopolyploidy, although the ratio was closer to the values expected for allohexaploids.

**Table 5:** The ratios of single-dose (SD) to multi-dose (MD) markers in the MK11/3 population.

	Observed	Expected	
		Allopolyploid	Autopolyploid
**Single-dose**	273	253.75	304.5
**Multi-dose**	133	152.25	101.5
**Markers in total**	406	406	406
**SD:MD**	0.67:0.33	0.625:0.375	0.75:0.25
$\chi_{0.95\,\left(d\,f=1\right)}^{2}$ **= 3.84**		3.894*	13.034*
***p*-value (α = 0.05)**		0.048*	0.0003*

The segregation ratios were compared with the theoretical proportions of SD to MD markers for allo- (0.625:0.375) and autopolyploidy (0.75:0.25) using the chi-square test (Silva et al., 1993). Significance is indicated with *, the critical value is $\chi_{0.95\,\left(d\,f=1\right)}^{2}$ = 3.84, and the *p*-values are given.

### AN ANALYSIS OF MARKER LINKAGE

Of the previously selected 245 SD markers, 80 markers were biparental and 165 were uniparental. These uniparental SD markers (81 for the maternal parent 541 and 84 for the paternal parent VZR) we used to identify markers in the coupling and the repulsion phases by a mapping approach. For 71 (32 for 541 and 39 for VZR) of the 165 markers, we showed linkage in coupling, whereas no markers were linked in repulsion and had LOD scores greater than 1.0. Therefore, the ratio of markers in coupling to those in repulsion was 1:0 (**Table 6**), as expected for an autopolyploid organism with a ploidy degree above tetraploidy.

**Table 6:** The ratios of the uniparental SD markers linked in coupling to those in repulsion for the MK11/3 population.

	Allopolyploid		Autopolyploid	
	Coupling	Repulsion	Coupling	Repulsion
**Observed**	71	0	71	0
**Expected**	35.5	35.5	71	0
$\chi_{0.95\,\left(d\,f=1\right)}^{2}$ **= 3.84**	43.6708*		0	
***p*-value (α = 0.05)**	3.89e^-11^*		1	

The obtained segregation ratio of 71 markers in coupling to 0 markers in repulsion was compared with the theoretical proportions for auto- (1:1) and allopolyploidy (1:0) using the chi-square test (Wu et al., 1992). Significance is indicated by *, the critical value was $\chi_{0.95\,\left(d\,f=1\right)}^{2}$ = 3.84, and the *p*-values are given.

## DISCUSSION

Based on their evolutionary origin and published cytological analyses, cultivated chrysanthemums have been mainly classified as allopolyploid plants (Watanabe, 1977, 1983; Li et al., 2011). However, several studies raised questions regarding whether the behavior of meiotic chromosomes is an appropriate indicator of the type of ploidy and therefore if the formation of bivalents or multivalents is a reliable indicator of whether a species is genetically an autopolyploid with tetrasomic inheritance or an allopolyploid with disomic segregation (Soltis and Rieseberg, 1986; Krebs and Hancock, 1989; Sybenga, 1996; Qu et al., 1998). With the advent of molecular markers as an informative genomic tool, Wu et al. (1992) and Silva et al. (1993) described effective methods based on SD markers to distinguish allopolyploids from autopolyploids. Therefore, we used molecular markers (AFLP, SSCP, and SSR,) to investigate the type of ploidy of cultivated chrysanthemums.

In total, we scored 406 polymorphic markers in the F1 MK11/3 population. Characteristic segregation ratios for allo- (e.g., 1:0 or 7:1) and autopolyploids (e.g., 4:1 or 9:1) have been established based on the type of ploidy of a genome. By using the chi-square test, the ratios of all of the segregating markers were compared to the theoretically expected segregation ratios. The vast majority of the markers (148) were not informative because they segregated in a ratio of 1:1, which is expected for a uniparental SD marker for allo- as well as autopolyploids. Additionally, 35 markers displayed skewed segregation ratios, which is a common phenomenon in plants (Mccouch et al., 1988; Gardiner et al., 1993; Wang et al., 1998) and has been reported for chrysanthemum (Zhang et al., 2010). Of the other markers, 13 segregated in a disomic manner (uniparental 3:1, 7:1 with some of them in linkage), which would be expected for an allopolyploid, whereas eight markers displayed a 4:1 ratio, which suggests a hexasomic inheritance between a duplex and a simplex marker. Indeed, it is difficult to reliably distinguish among several similar segregation ratios, as this requires a large number of individuals. Langton (1989) also described the hexasomic inheritance of the carotenoid pigmentation in chrysanthemums, but even this study was not considered as conclusive by the author himself because of conflicting results of Jordan and Reimann-Philipp (1983) on the inheritance of anthocyanin pigmentation. Also the analysis of the marker dosages, which revealed a 0.67 to 0.33 ratio of SD to MD markers, showed significant differences to the ratios expected for both, disomic (0.625:0.375) and hexasomic (0.75:0.25) inheritance (Silva et al., 1993; Silva and Sorrells, 1996; Ukoskit and Thompson, 1997).

Therefore, we analyzed the segregation patterns of three EST-SSRs in addition to the AFLP markers. For each marker, the alleles were inherited in all possible combinations and not independent of each other, as would be expected for fixed heterozygosity. This result indicates polysomic inheritance, as expected for autopolyploids. Therefore, it is very likely that the progenitors of cultivated chrysanthemums were phylogenetically closely related (Dai et al., 1998; Wang et al., 2002).

Furthermore, we did not detect any markers linked in repulsion in our mapping approach. This result also supports our hypothesis that chrysanthemums display polysomic inheritance. Two other published mapping approaches in chrysanthemums provide no information about the type of linkage of the mapped markers (Zhang et al., 2010, 2011). By increasing the number of markers, the mapping resolution could be improved, but this does not explain the lack of markers linked in repulsion in our study.

To summarize our marker results, two methods (segregation patterns of SSRs and the ratios for markers in coupling to those in repulsion) clearly showed a polysomic inheritance in chrysanthemums, as is characteristic of an autopolyploid. Nevertheless, some markers segregated in a disomic manner and the ratio of marker dosages was close to the expected ratio for disomic inheritance, but not significant. Therefore, the inheritance in chrysanthemum seems to be mainly polysomic with a random assortment of homologs, but there are a few loci with disomic inheritance as well due to a partial preferential pairing of chromosomes. This mixed inheritance has already been detected in cytological studies in chrysanthemum that reported the predominant formation of bivalent chromosomes and the occurrence of multivalent chromosomes, though only in a small proportion (Dowrick, 1953; Chen et al., 2009; Li et al., 2011). Such intermediates have also been described in strawberries (Lerceteau-Kohler et al., 2003), rapeseed (Udall et al., 2005), and yellow cress (Stift et al., 2008). Thus, we propose to classify cultivated chrysanthemums as segmental allopolyploids according to Stebbins (1947).

This change in classification is important for the breeding progress of chrysanthemums. If chrysanthemums were strict allopolyploids, the free combination of desirable alleles would not occur. In our study, we showed that most molecular markers were inherited in a polysomic manner. Therefore, the desirable alleles can be enriched in the gene pool independently of their subgenomic origins. Finally, the complex inheritance of ornamental traits in a segmental allo-hexaploid plant limits the effectiveness of marker-assisted selection, and phenotypic selection should be prioritized.

As Stebbins noted decades ago, it might be difficult to unambiguously determine the type of ploidy of an organism. In addition to cytological methods, molecular markers are useful tools with which to investigate the type of ploidy, and the combination of both approaches might be necessary to reveal the true type of ploidy. Based on the results of cytological studies, which report the predominant occurrence of bivalent chromosomes, a disomic inheritance was postulated for chrysanthemums. In contrast to these data, our analyses of molecular markers indicate a polysomic inheritance. Therefore, we suggest changing the classification of chrysanthemums from allopolyploid to segmental allopolyploid.

## Conflict of Interest Statement

The authors declare that the research was conducted in the absence of any commercial or financial relationships that could be construed as a potential conflict of interest.
